# Cervical Fracture Stabilization within 72 Hours of Injury is Associated with Decreased Hospitalization Costs with Comparable Perioperative Outcomes in a Propensity Score-Matched Cohort

**DOI:** 10.7759/cureus.244

**Published:** 2015-01-28

**Authors:** Zachary Medress, Robert T Arrigo, Melanie Hayden Gephart, Corinna C Zygourakis, Maxwell Boakye

**Affiliations:** 1 Department of Neurosurgery, Stanford University; 2 School of Medicine, Stanford University; 3 Department of Neurosurgery, University of California, San Francisco; 4 Department of Neurosurgery, University of Louisville

**Keywords:** spinal cord injury, cervical fusion, timing of surgery, surgical outcomes, spine trauma, health economics, propensity score modeling

## Abstract

Introduction: Prior studies have indicated that early decompression of traumatic cervical fractures can be performed safely and is associated with improved outcomes, though the economic impact of the timing of surgery in the American population has not been studied. After adjusting for patient, hospital, and injury confounders, we performed propensity score modeling (PSM) on a large clinical administrative database to determine associated costs depending upon timing of surgery for acute cervical fracture.

Methods: A total of 3,348 patients with surgically treated, traumatic, cervical fractures were identified. Patients were sorted into early (within 72 hours of admission) and late (beyond 72 hours) surgery groups. PSM was able to match 2,132 early and late surgery patients on age, comorbidity, expected payer, trauma severity, hospital type, urgent admission, and surgical approach. Perioperative complications, mortality, and resource utilization were assessed.

Results: Late surgery was more frequently associated with increased age, more comorbidities, higher ICISS score, and non-private insurance. Following PSM matching, there were no significant, preoperative differences between early and late surgery groups. Surgery performed after 72 hours was associated with an increase in in-hospital complications (OR=1.3). The early surgery group was associated with decreased length of stay (11 days vs. 16 days, p <0.0001) and hospital charges ($237,786 v. $282,727, p <0.0001).

Conclusions: After controlling for potential confounding differences through PSM matching and multivariate analyses, we found late surgery independently associated with increased in-hospital complications, length of stay, and hospital resource utilization. These data suggest surgery within 72 hours may decrease resource utilization without a corresponding increase in postoperative morbidity.

## Introduction

There is conflicting evidence regarding the optimal timing of surgical intervention for traumatic cervical spine fractures, creating a dilemma for treating surgeons. Discrepancies between studies addressing the safest time for surgical intervention relates to complex medical conditions, varying etiologies of the fractures, and a wide range of potential neurologic deficits encompassed within a single diagnosis. The varied clinical presentation, combined with a small patient cohort, makes it difficult to control for all of the potential variables influencing the timing of surgical intervention and outcomes. Several animal studies suggest that early surgical decompression leads to better neurological outcomes by preventing deleterious secondary effects of the initial spinal cord injury, including edema, hemorrhage, ischemia, and excitotoxic cell death [1-4]. Outcomes in human studies have been mixed, ranging from potential increased risk [5-6], to no difference [7], to improved outcomes with decreased cost [8]. This lack of consensus in the literature led to insufficient evidence to define the optimal timing of surgical intervention for cervical spine fractures [9-11].

Early fracture stabilization is associated with improved neurologic recovery [12], decreased length of stay, and decreased costs [13], although the cost savings associated with early surgery have not been quantified in the American population. Recently, the Surgical Timing in Acute Spinal Cord Injury Study (STASCIS), a large, multi-center, prospective cohort study, indicated that early decompression can be performed safely and is associated with improved neurological recovery at six months after surgery [12]. However, STASCIS did not evaluate the economic effect or length of hospital stay associated with timing of surgery. A Canadian study showed that early surgery within 24 hours is associated with decreased costs and length of stay [13], though the cost savings of early surgery have not been described in the American population. In the United States, spinal cord injury incidence has been estimated at 56.4 per million adults, with an incidence of 87.7 cases per million in older adults and is rising [14]. Given its high cost of care in the acute and chronic periods, spinal cord injury represents a large financial burden on the American healthcare system. Greater knowledge of the degree of cost-savings associated with early surgery is important in providing high quality and cost-effective care.

To address these issues, we identified within the California State Inpatient Databases (CA SID) a large, multi-center cohort of patients with traumatic cervical fractures, with or without spinal cord injury that underwent cervical fusion. Propensity-score modeling (PSM) using a 1:1 optimal matching algorithm controlled for many confounding factors within this diverse group of patients, and allowed focus on the potential influence of early (within 72 hours of admission) or late surgical intervention on hospital charges, length of stay, and in-hospital complication rates.

Consent was formally obtained or waived for all subjects present within this study.

## Materials and methods

### Data source and study criteria

The California State Inpatient Databases (CA SID) are produced and distributed by the Agency for Healthcare Research and Quality’s Healthcare Cost and Utilization Project (HCUP). These databases include discharge data in over 95% of hospitalizations at both community and non-community (e.g. federal) hospitals within California [15]. The reliability of CA SID has previously been demonstrated [16]. Patients with surgically treated cervical fractures, with or without spinal cord injury, were identified by International Classification of Disease, Ninth Revision (ICD-9) codes in 2003 to 2011 CA SID. A notable advantage of CA SID is the presence of an encrypted linking variable that allows for following a single patient across multiple hospitalizations and years.

We identified hospitalizations for all adults (age ≥18 years) undergoing surgical treatment for traumatic cervical fractures from 2003 to 2011. Cases were included only if surgery occurred on or after the day of admission and if the time from hospital admission to surgery was known. Primary ICD-9 procedure codes were cervical fusion with either an anterior (81.02) or posterior approach (81.03), and primary ICD-9 diagnosis codes for open or closed cervical fracture with or without spinal cord injury (805.0, 805.1, 806.0, 806.1). Patients meeting these criteria were separated into two groups corresponding to whether surgery occurred within 72 hours of hospital admission, defined as ‘early’ surgery, or after 72 hours of admission, defined as ‘late’ surgery. As an alternative classification of patients as having surgery within (early) or beyond 24 hour of admission (late) has previously been used [17-18], we repeated our results with a secondary classification utilizing this time point.   

### Recorded data

Data were recorded for each patient on *a priori* determined parameters: admission from emergency department, age at admission, insurance provider (private v. Medicare v. all other, including Medicaid and uninsured/self-pay), ICD-9 diagnosis and procedure codes, number of days from admission to surgery, discharge status [routine (to home) vs. non-routine (to anywhere other than home)], length of stay, hospital charges, and inpatient mortality. A comorbidity score was assigned to each patient based on a count of the number of Elixhauser comorbidities identified among each patient’s ICD-9 diagnosis codes (excluding the Elixhauser grouping for paralysis, which was treated as a sequelae of the cervical fracture) [19-20]. Degree of traumatic injury was quantified using the ICD-9 Based Injury Severity Score (ICISS) [21], and survival risk ratios published by the American College of Surgeons [22] to estimate the probability of survival for each patient given the set of assigned diagnosis codes. The ICISS score was subtracted from 1 and multiplied by 100 to produce a 0 (certain survival) to 100 (certain death) probability of death corresponding to injury severity (the injury severity score). In-hospital postoperative complications were identified by ICD-9 diagnosis code as follows: renal (584, 997.5), cardiac (997.1, 410.0 – 410.91), neurologic (997.00 – 997.09), deep-vein thrombosis or pulmonary embolism (415.1, 415.11, 415.19, 451.1, 451.11, 451.19, 451.2, 451.81, 451.9, 453.4, 453.40, 453.41, 453.42, 453.8, 453.9), pulmonary (507.0, 518.4, 518.5, 518.81, 518.82, 997.3, 997.31, 997.39), infection (038, 320, 510, 513.1, 519.2, 590.1, 590.80, 683), and wound complications (998.1 – 998.7, 998.9). Where appropriate, the CA SID’s “diagnosis present on admission” indicator was used to distinguish a comorbidity from a complication (e.g. pulmonary embolism) [23]. Hospital costs were estimated from hospital charges using the cost-to-charge data provided by HCUP for cases from 2006 to 2009, and adjusted to 2009 US dollars. Hospital charges are what the hospital billed, whereas hospital costs are what the actual costs were, and represent a better indicator of actual resource utilization.

### Statistical analysis

Propensity score modeling (PSM) is a statistical method that can be applied to retrospective data in an attempt to simulate a randomized controlled trial [24-25]. All of the relevant, known, and measured variables in our study (e.g. age) were tested for statistical significance with the outcome (time to surgery) using standard univariate analysis methods (T-test for continuous data, Chi-square or Fisher exact test – as appropriate – for categorical data). Variables significantly associated with time to surgery (P < 0.05) were then used in an optimal 1:1 PSM matching [26-27] to balance these variables between treatment groups. The maximum allowable propensity score distance between each matched early: late surgery pair was set to 0.01. The quality of the PSM match was assessed both graphically (comparing propensity score histograms for early vs. late surgery) and statistically (standardized differences for each balancing variable were computed; a score of 0.10 or less was considered well balanced) [28-29]. All balancing variables used in the match (age, trauma severity, comorbidities, ED admission, ICISS score, expected payer, hospital, and surgical approach) passed both visual and statistical quality-checks (Table 2). Thus, from 3,348 surgically treated spinal cord injury patients from CA SID, a subset of 2,132 patients could be well matched and included in the PSM matched cohort used in this study.

Perioperative outcomes, mortality, and resource utilization (also chosen *a priori*) were assessed in the two treatment groups. Univariate analysis was performed on the matched cohort to identify variables significantly associated with in-hospital complications using T-test, Chi-square, or Fisher exact test as appropriate. Variables with P < 0.15 were included in a multivariate logistic regression model to determine whether timing of surgery predicted perioperative complication rate after PSM match balancing between treatment groups while controlling for other factors. All analyses were performed using SAS software (version 9.4; SAS Institute, Inc., Cary, NC) on Windows 7 64-bit Professional.  In this study, P-values less than 0.05 were interpreted as statistically significant. 

## Results

We identified 3,348 CA SID patients who underwent surgical treatment for cervical fracture, with or without spinal cord injury, between 2003 and 2011. Before PSM matching, the mean age of patients in the early surgery group (within 72 hours) was 48.9 years, versus 53.3 years for patients in the late surgery group. In the unmatched cohort, the early treatment group had fewer comorbidities than the late treatment group (1.28 vs. 1.82; P<0.0001) and a lower trauma severity code (5.3% vs. 6.5%; P<0.0001) at the time of hospital admission. Additional imbalances between treatment groups were identified (Table 1); the presence of spinal cord injury was not significantly different between the two groups and was therefore not included in the matching. PSM matching was then performed, and we selected 1,066 pairs of patients treated before and after 72 hours for a total of 2,132 patients in the PSM-matched cohort. Successful PSM matching was confirmed by visual inspection of histograms and calculated standardized differences for each balancing variable (Table 2).

**Table 1:** Patient and Surgical Characteristics for Entire Cohort, Overall and by Time to Surgery ^a ^Chi-square or Fisher Exact test comparing the ratio of patients treated within 72 hours versus patients treated beyond 72 hours for each row variable (e.g., ‘Were females more likely to be treated with later surgery than males?’). P < 0.05 is significant. ^b ^Presented as mean [Q1, Q3].

	Overall		Treated Within 72 Hours		Treated Beyond 72 Hours		P-value^a^	
	Cases	(%)	Cases	(%)	Cases	(%)		
Overall	3,348	(100)	2,249	(100)	1,099	(100)		
Admitted from ED							<0.0001	*
Yes	2,311	(69.0)	249	(22.7)	788	(35.0)		
No	1,037	(31.0)	850	(77.3)	1,461	(65.0)		
Age							<0.0001	*
Under 65	2,422	(72.3)	1,684	(74.9)	738	(67.2)		
65 and older	926	(27.7)	565	(25.1)	361	(32.9)		
Elixhauser comorbidity group							<0.0001	*
Zero	1,257	(37.5)	941	(41.8)	316	(28.8)		
One	839	(25.1)	567	(25.2)	272	(24.8)		
Two	518	(15.5)	327	(14.5)	191	(17.4)		
Three	316	(9.4)	187	(8.3)	129	(11.7)		
Four or more	418	(12.5)	227	(10.1)	191	(17.4)		
Expected payer							<0.0001	*
Private	1,414	(42.2)	1,050	(46.7)	364	(33.1)		
Medicare	871	(26.0)	525	(23.3)	346	(31.5)		
Other including Medicaid	1,063	(31.8)	674	(30.0)	389	(35.4)		
Sex							0.5504	
Female	1,036	(31.1)	703	(31.4)	333	(30.3)		
Male	2,300	(68.9)	1,535	(68.6)	765	(69.7)		
Urban-rural continuum							0.0059	*
Large metropolitan area	2,304	(69.9)	1,510	(68.1)	794	(73.4)		
Small metropolitan area	838	(25.4)	600	(27.1)	238	(22.0)		
Micropolitan or smaller	156	(4.7)	106	(4.8)	50	(4.6)		
ICISS-based probability of death^b^	5.7%	(1.6,6.1)	5.3%	(1.2, 5.7)	6.5%	(1.8, 7.2)	<0.0001	*
Surgical approach							0.0129	*
Anterior (ICD-9, 81.02)	2,129	(63.6)	1,463	(65.1)	666	(60.6)		
Posterior (ICD-9, 81.03)	1,219	(36.4)	786	(35.0)	433	(39.4)		
Spinal cord injury							0.5985	
Yes (ICD-9, 806)	974	(29.1)	661	(29.4)	313	(28.5)		
No or unknown (ICD-9, 805)	2,374	(70.9)	1,588	(70.6)	786	(71.5)		

**Table 2:** Standardized Differences of Balancing Variables used for PSM Matching Standardized differences for each balancing variable used during propensity score modeling. Values less than 0.10 indicate balance was achieved between treatment groups.

Balancing Variable	Standardized Difference
Admitted from emergency department	0.032
Age group	0.002
Elixhauser comorbidity score	0.004
Expected payer	0.027
Injury severity score	0.025
Surgical approach	0.033
Known spinal cord injury	0.029
Urban-rural continuum	0.012

Patient, hospital, and injury data for the PSM matched cohorts are presented in Table 3. No statistically significant differences between the late and early surgery cohorts were seen after matching. Morbidity, mortality, and resource utilization are presented in Table 4. The overall in-hospital complication rate was 27.7%, with an increase in complications associated with late surgery (29.6% vs. 25.7%; P<0.05). When sub-stratifying in-hospital complications by system, the early surgery group showed decreased incidences of postoperative infections (1.8% vs. 3.6%; P<0.05) and renal complications (3.0% vs. 5.2%; P<0.02). Furthermore, compared to the late surgery group, early surgery patients had shorter hospitalizations (10.9 days vs. 15.7 days; P<0.0001) and incurred lower hospital charges ($237,786 vs. $282,727; P<0.0001). Hospital costs were available for cases from 2006 through 2009 and showed decreased resource utilization with early surgery ($63,065 vs. $77,049; P=0.0007). There was no significant difference in in-patient mortality.

**Table 3:** Patient Characteristics for PSM Matched Cohort, Overall and by Time to Surgery ^a ^Chi-square or Fisher Exact test comparing the ratio of patients treated within 72 hours versus patients treated beyond 72 hours for each row variable (e.g., ‘Were females more likely to be treated with later surgery than males?’). P < 0.05 is significant. ^b ^Presented as mean [Q1, Q3].

	Overall		Treated Within 72 Hours		Treated Beyond 72 Hours		P-value^a^	
	Cases	(%)	Cases	(%)	Cases	(%)		
Overall	2,132	(100)	1,066	(100)	1,066	(100)		
Admitted from ED							0.4990	
Yes	1,656	(77.7)	835	(78.3)	821	(77.0)		
No	476	(22.3)	231	(21.7)	245	(23.0)		
Age							1.0000	
Under 65	1,429	(67.0)	715	(67.1)	714	(67.0)		
65 and older	703	(33.0)	351	(32.9)	352	(33.0)		
Elixhauser comorbidity group							0.9051	
Zero	610	(28.6)	300	(28.1)	310	(29.1)		
One	539	(25.3)	273	(25.6)	266	(25.0)		
Two	382	(17.9)	196	(18.4)	186	(17.5)		
Three	255	(12.0)	130	(12.2)	125	(11.7)		
Four or more	346	(16.2)	167	(15.7)	179	(16.8)		
Expected payer							0.8046	
Private	706	(33.1)	346	(32.5)	360	(33.8)		
Medicare	678	(31.8)	341	(32.0)	337	(31.6)		
Other including Medicaid	748	(35.1)	379	(35.6)	369	(34.6)		
Sex							0.2427	
Female	666	(31.3)	345	(32.5)	321	(30.1)		
Male	1,460	(68.7)	716	(67.5)	744	(69.9)		
Urban-rural continuum							0.7776	
Large metropolitan area	1,546	(72.5)	768	(72.1)	778	(78.0)		
Small metropolitan area	489	(22.9)	251	(23.6)	238	(22.3)		
Micropolitan or smaller	97	(4.6)	47	(4.4)	50	(4.7)		
ICISS-based probability of death^b^	6.2%	(1.7,7.1)	6.1%	(1.7, 7.1)	6.3%	(1.8, 7.1)	0.5698	
Surgical approach							0.4754	
Anterior (ICD-9, 81.02)	1,321	(62.0)	669	(62.8)	652	(61.2)		
Posterior (ICD-9, 81.03)	811	(38.0)	397	(37.2)	414	(38.8)		
Spinal cord injury							0.5353	
Yes (ICD-9, 806)	620	(29.1)	317	(29.7)	303	(28.4)		
No or unknown (ICD-9, 805)	1,512	(70.9)	749	(70.3)	763	(71.6)		

**Table 4:** Outcomes of PSM Matched Cohort ^a ^Chi-square, Fisher Exact, or T-Test, as appropriate. P < 0.05 is significant. ^b ^Includes only cases from 2006 to 2009, the years for which cost-to-charge data was available.

	Overall	Treated Within 72 Hours	Treated Beyond 72 Hours	P-value	^a^
In-hospital complications (%)	27.7	25.7	29.6	0.0471	*
Cardiac	2.1	1.7	2.5	0.2277	
Infection	2.7	1.8	3.6	0.0150	*
Neuro	1.0	1.0	0.8	0.6461	
Pulmonary	21.6	20.8	22.3	0.4297	
Renal	4.1	3.0	5.2	0.0156	*
VTE	3.9	3.4	4.5	0.2205	
Wound	2.7	2.4	3.0	0.5060	
In-hospital mortality (%)	3.4	3.8	3.0	0.4015	
Non-routine discharge (%)	54.2	52.1	56.3	0.0558	
Length of stay, mean (days)	13.3	10.9	15.7	< 0.0001	*
Total charges, mean ($)	$260,326	$237,786	$282,727	< 0.0001	*
Total hospital cost^b^, mean ($)	$260,326	$63,065	$77,049	0.0007	*

In order to better delineate the demographic and perioperative factors associated with in-hospital complications following cervical fusion, logistic regression was performed on PSM matched patient data (Table 5). Increasing age, comorbidity burden, trauma severity code, male sex, documented spinal cord injury at presentation, and delayed surgery were all associated with increasing odds of in-hospital complications. The strongest predictor of in-hospital complications was documentation of spinal cord injury (OR=3.169, 95% CI, 2.461 to 4.079). After PSM matching, the effect of delayed surgery beyond 72 hours was associated with a 27.3% increase in odds of in-hospital complications versus surgery performed within 72 hours.

**Table 5:** Logistic Regression on Presence of In-Hospital Complications after Cervical Fusion, PSM Matched Cohort * P < 0.05 is significant C-statistic: 0.811

	Group	Odds Ratio	95% CI	
Age	Single point increase	2.816	[1.894, 4.186]	*
Expected payer	Medicare v. private insurance	0.671	[0.445, 1.011]	
	Other including Medicaid v. private Insurance	0.947	[0.709, 1.265]	
Elixhauser comorbidity score	Single point increase	1.581	[1.476, 1.695]	*
ICISS probability of death	Single percentage point increase	1.062	[1.046, 1.078]	*
Sex	Male v. female	1.463	[1.143, 1.873]	*
Spinal cord injury	Yes (ICD-9, 806) v. no/unknown (ICD-9, 805)	3.169	[2.461, 4.079]	*
Surgical approach	Anterior (ICD-9, 81.02) v. posterior (ICD-9, 81.03)	0.916	[0.729, 1.152]	
Time to surgery	Over 72 hours v. within 72 hours	1.273	[1.019, 1.589]	*
Urban-rural continuum	Large metropolitan v. nicropolitan	1.129	[0.644, 1.982]	
	Small metropolitan v. micropolitan	1.222	[0.675, 2.213]	

Prior studies have defined “early” surgery as being prior to 72 hours [8, 30-31]; however, alternative cutoff points could be used [17-18]. To account for this variability, we repeated the entire analysis using 24 hours as the cut off for early surgery, and found similar results. Specifically, after PSM matching, in-hospital complications, and hospital resource utilization remained statistically significantly elevated in the late surgery group (>24 hours). In the multivariate model for the 24-hour time point, diagnosis of spinal cord injury was again the strongest predictor of in-hospital complication (OR=4.266; 95% CI, 3.114 to 5.844), and late surgery was again associated with an increased odds of complication (OR=1.484; 95% CI, 1.116 to 1.974).

## Discussion

In this retrospective study, we sampled from a large, public administrative database to assess the impact of the timing of surgery following traumatic cervical fracture on in-hospital complication rates, mortality rates, and resource utilization. Diverse demographics, complicated medical histories, and varying levels of multisystem injury severity can confound studying outcomes of patients undergoing cervical fractures for spinal cord injury. PSM enabled us to balance known, measured, preoperative factors that were significantly different between time-to-surgery groups. Spinal cord injury patients in this study were not randomized to either late or early surgical intervention; therefore, there may have been underlying bias determining the timing of surgery. Patients with private insurance were more likely to receive surgery within 72 hours, whereas patients with Medicaid, Medicare, and other expected payers were more frequently in the late surgery cohort. Advanced age (>65 years), increased comorbidities, and a higher trauma severity score was more frequently associated with surgery after 72 hours, perhaps attesting to the overall complexity of the patients’ medical situation and appropriateness for surgery. 

When controlling for medical comorbidities, trauma severity, and age, among other potential confounding factors, the PSM matched cohorts showed cervical fusion performed within 72 hours of hospital admission was independently correlated with fewer in-hospital complications, shorter hospital stays, and lower healthcare resource utilization. There was no associated increase in mortality rate with early surgery. Our study, which includes patients from a diverse set of community and federal hospitals across California, is the largest to date that addresses the question of when patients suffering from acute cervical spinal fracture should undergo cervical decompression surgery. Our results build on previous, smaller, retrospective studies which have shown that early surgery is associated with shorter hospital stays [17], lower incidence of postoperative complications [8, 32-33], and decreased costs [13] (Table 6). In contrast to our results, two previous prospective studies did not find a significant impact of time-to-surgery on outcomes. Specifically, a prospective, randomized study in 1997 by Vaccaro, et al. of 64 cervical spine patients showed no difference in the length of postoperative hospital stay between patients who were operated on early (< 72 hours after injury) versus late [7]. Notably, postoperative complication rates were not measured in this study.

More recently, STASCIS demonstrated no difference in complication rates between early (<24 hours) and late treatment group but did not evaluate the effect of timing of surgery on healthcare utilization [12]. Patients participating in STASCIS were not randomized to early or late surgery groups, leading to a higher average age in the late surgery group. Likewise, our study found the late surgery group to contain a greater proportion of people older than 65 years. Although retrospective, our study benefits from PSM matching to generate an equal balance between groups when considering age, expected payer, injury severity score, surgical approach, and urban-rural continuum. Nonetheless, the present study does have shortcomings and limitations. Although we considered stratifying patients according to fracture type, including destabilizing versus non-destabilizing fractures, the CA SID does not include information about fracture type, which is an important clinical feature that likely influences decision-making about timing of surgery. In addition, hospital charges associated with intensive care unit (ICU) care, a metric that deserves study in future research given the high costs associated with ICU-level care, were not available for analysis using the CA SID. The time point used to differentiate early versus late surgery is controversial, and a number of cut-off points have been used in prior studies (Table 6). Although patients in our study were equally matched to the Elixhauser comorbidity score and ICISS probability of death, delayed surgery after 72 hours may reflect factors that were not accounted for in this study that may prompt a surgeon to delay surgery, such as the presence of non-destabilizing fractures or other factors that impact prognosis. Lastly, our study suffers from shortcomings that are inherent in outcomes research using large administrative databases, and we recognize that causal association between timing of surgery and resource utilization cannot be definitely made using a retrospective administrative database.

**Table 6:** Summary of Previous Studies on the Timing of Cervical Fusion following Spinal Cord Injury

First Author	Year	# of Patients	Study Design	Inclusion Criteria	Cut-off Time	Findings
Aebi M [32]	1986	100	Retrospective	Cervical spinal injuries treated operatively	N/A	Early surgery associated with shorter ICU stay, lower incidence of pulmonary complications, and lower cost of treatment.
Croce MA [8]	2001	291	Retrospective	Spine fractures due to blunt trauma admitted to an urban level 1 trauma center	3 days	Early surgery associated with shorter ICU stay, lower incidence of pulmonary complications, and lower cost of treatment.
Fehlings MG [12]	2012	313	Prospective	Adults (age 18-60) with cervical SCI	1 day	Early surgery associated with higher rate of ≥ 2 grade improvement in ASIA Impairment Scale grade at 6 months follow-up. No significant difference in complication rate between early and late treatment group.
Heiden, JS [5]	1975	356	Retrospective	Operated and nonoperated patients with complete and incomplete cervical myelopathies	7 days	Patients with complete lesions did not benefit from early treatment. Early anterior cervical fusion for patients with complete sensorimotor paralysis was associated with increased pulmonary morbidity.
Levi L [17]	1991	103	Retrospective	Cervical spine trauma treated with anterior decompression	1 day	Early surgery associated with shorter hospital stay and fewer respiratory procedures required.
Mac-Thiong [13]	2012	477	Retrospective	Acute traumatic spinal cord injury at any spinal cord level with evidence of cord compression	1 day	Early surgery associated with decreased costs and length of stay.
Marshall LF [6]	1987	283	Prospective	Spinal-cord injured patients	5 days	Neurological deterioration observed in cervical spine-injured patients undergoing early surgery, but not seen in the late surgery group.
Mirza SK [31]	1999	43	Retrospective	Acute cervical spinal injury neurologic deficit treated surgically	3 days	Early vs late surgery not associated with changes in complication rate. Early surgery may improve neurologic recovery.
Papadopoulous SM [34]	2002	91	Prospective	Acute traumatic spinal cord injury	“Immediate”	Immediate spinal column stabilization associated with improved neurologic outcome.
Pollard ME [18]	2003	412	Retrospective	Traumatic incomplete cervical spinal injuries	1 day	Neurologic recovery not associated with timing of surgery.
Sapkas GS [30]	2007	67	Retrospective	Fracture and fracture-dislocations at C3-7 treated surgically	3 days	No statistically significant difference in final neurological outcome between early and late treatment groups.
Wagner FC [33]	1982	44	Retrospective	Closed cervical spine and spinal cord injury at C3-7	2 days	Early surgery associated with shorter ICU stay, lower incidence of pulmonary complications, and lower cost of treatment.
Vaccaro AR [7]	1997	123	Randomized prospective	Traumatic spinal cord injuries at C3-T1	Early surgery: 72 hours, late surgery: >5 days	No significant difference in length of intensive care stay, improvement in American Spinal Injury Association grade, or motor score.

In our study, patients treated surgically 72 hours after hospital admission remained in the hospital on average five days longer than matched patients undergoing early surgery, and incurred total charges (costs) of $44,941 ($13,984), more than patients treated with early surgery. This greater healthcare resource utilization observed in the late treatment group is most likely due to a combination of increased hospital stay and increased incidence of in-hospital complications. Other clinical factors known to significantly impact resource utilization in the setting of acute cervical spinal cord injury, such as injury severity score or comorbidity score, were balanced between the early and late surgery groups using PSM matching and, therefore, were not likely to contribute to the increase in resource utilization. The presence or absence of spinal cord injury was associated with an increase in risk of postoperative morbidity, but not significantly different in occurrence between the two cohorts. Our data support previous work indicating early surgery following cervical spine injury leads to decreased resource utilization [8, 32-33]. This finding is particularly relevant in the era of bundled payment systems in which physicians and hospitals are compensated as a composite sum for episodes of care rather than a fee-for-service model. In this setting, neurosurgeons will be forced to make clinical decisions to provide care at a lower cost as long as quality and outcome are not affected. Ultimately, medical complexity and decisions regarding the timing of surgery cannot be completely captured by large, multi-center databases and coding. Our study provides a reflection on the potential cost savings of early intervention under the appropriate clinical scenario.

## Conclusions

We conducted a carefully controlled survey of a statewide, clinical administrative database to assess whether late surgery following cervical fracture was associated with increased resource utilization in the American population. Late surgery was more frequently associated with increased age, more comorbidities, higher ICISS score, and non-private insurance expected payer. After controlling for these potential confounding differences between the late and early surgery cohorts through PSM matching and multivariate analyses, we found late surgery was independently associated with increased in-hospital complications. Late surgery was correlated with increased hospital resource utilization in the form of longer hospital stays and increased hospital charges. The decision when to operate on a patient with a traumatic cervical fracture must be tailored to the individual needs of the patient, resources of the hospital and operating room staff, and complexity of the patient’s neurological and clinical situation. When controlling for these important factors, these data would suggest surgery within 72 hours on a medically appropriate patient may decrease resource utilization, without a corresponding increase in postoperative morbidity.
